# A new medical device applied in a case of acute fecal impaction with overflow diarrhea: a case report

**DOI:** 10.1186/s13256-024-04407-4

**Published:** 2024-03-06

**Authors:** Qin Huang, Fan Zheng, Hongxia Wang, Yong Yang, Chun Ma, Likun Zhu

**Affiliations:** 1 The Affiliated Hospital of Southwest Medical University, Luzhou, 646000 China; 2 Anorectal Department, Deyang People’s Hospital, Deyang, 618000 China; 3 School of Industrial Technology, Universiti Sains Malaysia, Penang, 11800 Malaysia; 4 General Department, Deyang People’s Hospital, Deyang, 618000 China; 5 Gastrointestinal Department, Deyang People’s Hospital, Deyang, 618000 China; 6 Radiology Department, Deyang People’s Hospital, Deyang, 618000 China

**Keywords:** Fecal impaction, Overflow diarrhea, Emergency, Medical device

## Abstract

**Background:**

Fecal impaction is a digestive system disease, that is most common in the elderly population and becomes more prevalent with increasing age. Manual removal can successfully remove the impaction in 80% of fecal impaction cases. In severe cases, endoscopy and surgery may be necessary.

**Case presentation:**

A 78-year-old Han Chinese man living in a nursing home was diagnosed with fecal impaction; his initial symptom was overflow diarrhea, which is a rare occurrence with regard to fecal impaction. Nevertheless, we were able to effectively treat this situation by employing a new medical device that presents a novel method for addressing fecal impaction.

**Conclusion:**

Early identification of fecal impaction with atypical symptoms is crucial to provide proper emergency management. A safe and noninvasive treatment method, especially for elderly patients with fecal impaction, should be chosen.

## Introduction

Fecal impaction (FI) is a digestive disease that occurs when a substantial amount of hard feces accumulates in the intestine and cannot be expelled [1, 2]. This condition is prevalent in all age groups, but elderly people in nursing homes are especially vulnerable, with approximately 50% experiencing it annually [3, 4]. FI is a leading cause of hospital admissions and increases morbidity and mortality [5]. Early identification and treatment of FI are crucial to prevent further harm. However, some atypical symptoms of FI, such as overflow diarrhea, may not be correctly identified initially [6, 7].

In mild cases, enema and osmotic laxatives, such as polyethylene glycol, are effective treatments; however, in severe or treatment-resistant cases, more invasive alternatives may be required [1, 8–10]. For elderly patients, surgery may cause further damage. Although endoscopy is a less invasive technique, it still carries the risk of bleeding and perforation [9]. Herein, we report a case of FI with overflow diarrhea as the initial symptom. We were able to effectively treat this situation by employing a new medical device that has not yet been widely introduced in the field.

## Case report

**Fig. 1:**
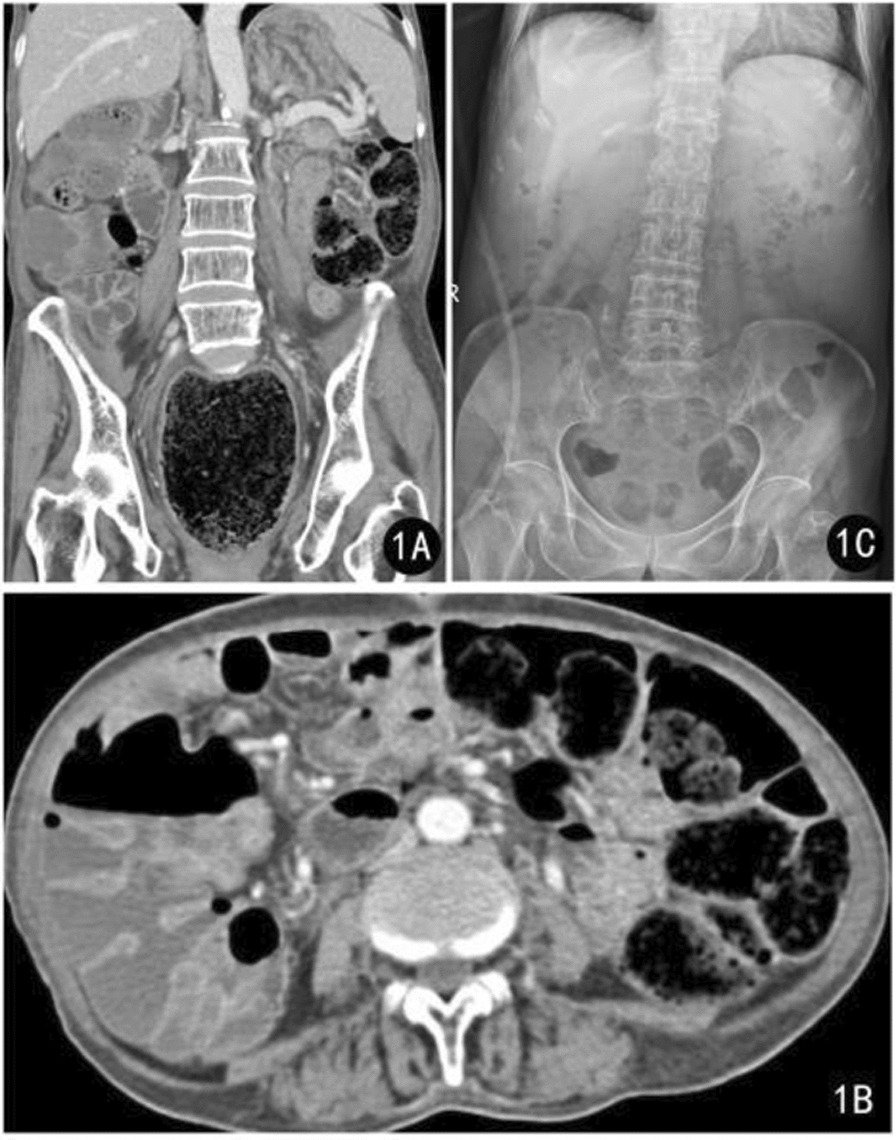
Abdominal imaging before and after case treatment

A 78-year-old Han Chinese man presented to the emergency department with a complaint of overflow diarrhea for 2 days. The patient had yellow loose stool discharge 2 days prior. Despite receiving medical treatment at the community health service center, he still experienced abdominal pain, bloating, and an inability to eat, coupled with continued discharge of yellow loose stool. The patient denied anal distention, urinary incontinence, fever, and other symptoms. The patient reported a medical history of coronary heart disease and lumbar degeneration. He had been residing in a nursing home for a long time without taking any medication lately. Additionally, his mobility was restricted to the bed or a wheelchair. The patient’s vital signs were normal. However, the abdomen was slightly swollen, and there was tenderness in the left lower quadrant with a cord-like induration. During the anal finger examination, a large accumulation of dry and fecal matter was found 3 cm into the anus. The heart, lungs, and nervous system showed no positive signs in the assessment. Routine tests for blood, urine, and stool, as well as liver and kidney function, showed no abnormalities. An abdominal computed tomography (CT) scan revealed an abundance of fecal matter in the rectum colon, and the proximal colon had significant dilation with an air-fluid surface in the ascending colon (Fig. 1a, b).

**Fig. 2:**
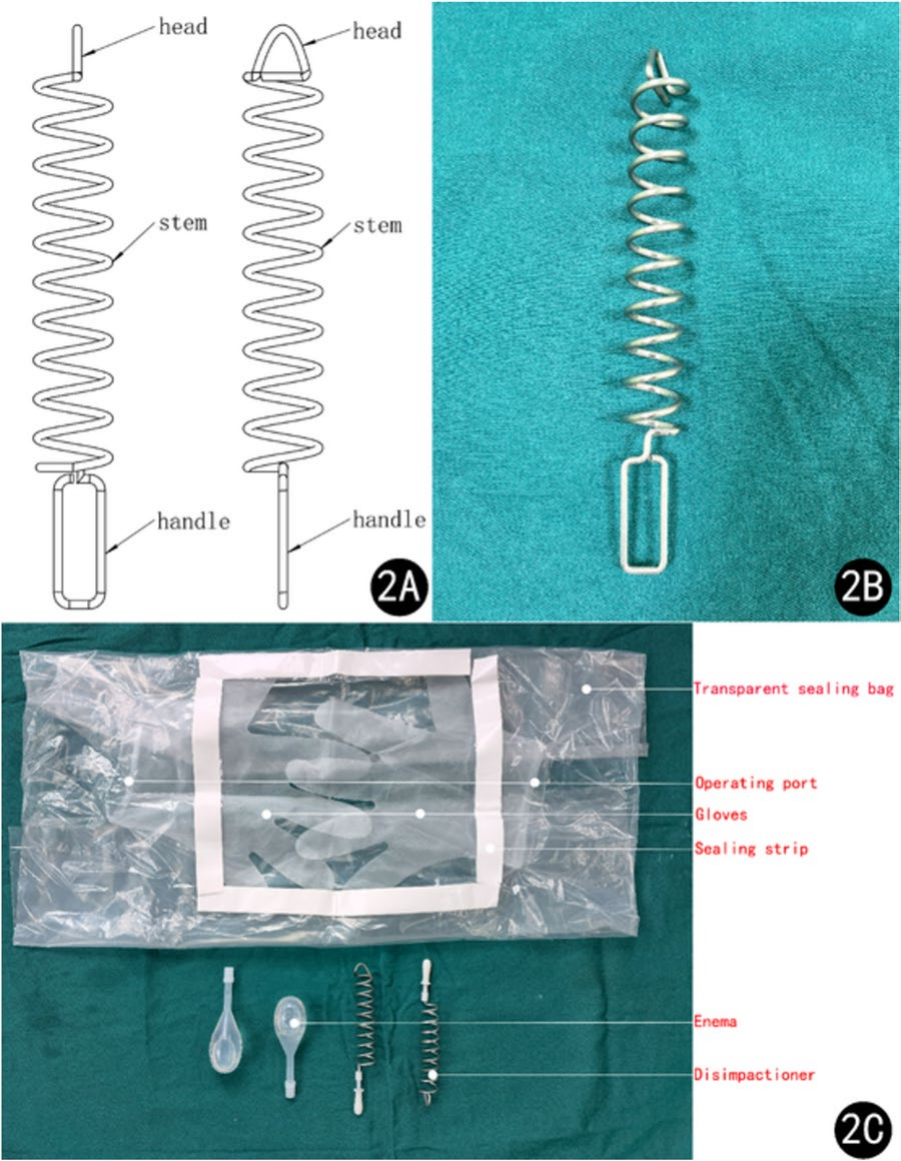
Disimpactioner, transparent sealing bag, and operational components

**Fig. 3:**
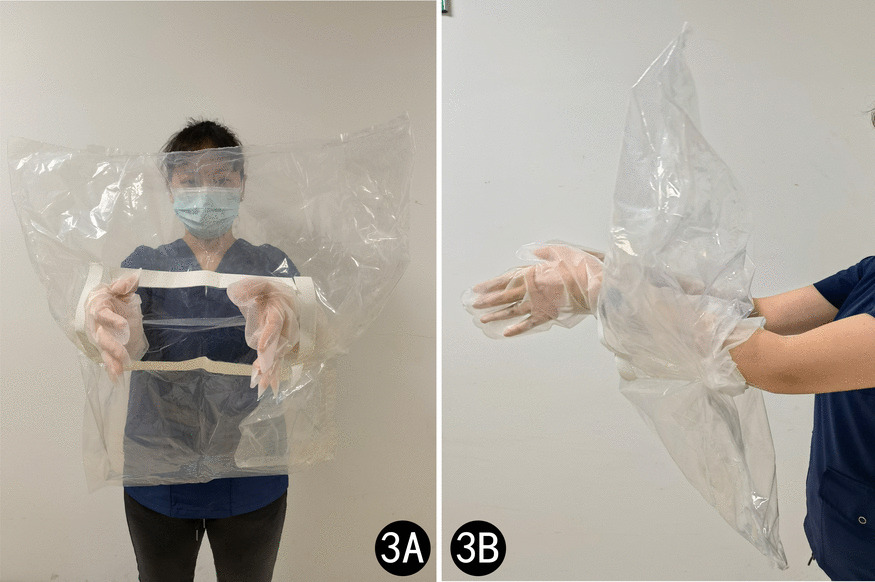
Schematic diagram of the use of transparent sealing bags

**Fig. 4:**
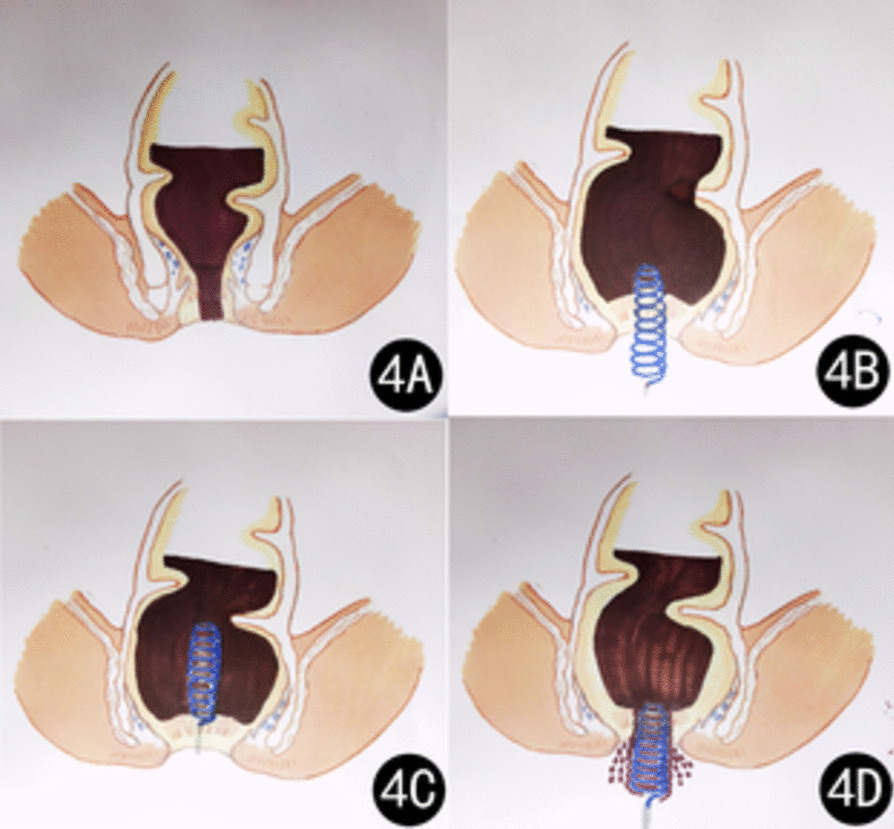
Schematic diagram of the Disimpactioner

The patient was diagnosed with FI. Despite attempting manual disimpaction, enema, and other methods, we were unsuccessful in relieving the condition. After assessing the patient’s stable vital signs and finding no indication of bleeding in their intact rectal mucosa, we decided to employ a new medical device (Disimpactioner, invention patent number: ZL201610548307.4, production license number: 18230601) for treatment. The Disimpactioner has a spiral shape and is made of disposable stainless-steel wire. It consists of a head, a stem, and a handle, with a dedicated transparent sealing bag equipped for the procedure. The transparent sealing bag consists of a sealing bag, a sealing strip, an operating port, and gloves connected to the operating port (Fig. 2). The patient was placed in a lateral position during the procedure. Upon encapsulating all operational components within the hermetic pouch, the practitioner proceeds to unseal four closure strips and affix the hermetic pouch onto the perianal region, ensuring centralized alignment with the anus. Subsequently, the operator inserts both hands into the integrated gloves via the operational port on the sealing bag, facilitating entry into the procedural field (Fig. 3). Following lubrication of the Disimpactioner, anus, and anal canal with liquid paraffin-soaked cotton balls, the separator is methodically and delicately rotated while being introduced into the anal orifice. Once the spiral body of the rod was fully inserted, it was slowly pulled, along with the decomposed fecal mass, out of the anus and placed in a sealed bag (Fig. 4). This procedure was repeated three times. Finally, 60 mL of enema was injected into the rectum. After a waiting period of 5–8 minutes, the patient discharged residual feces from the rectum.

Following treatment, the patient experienced immediate relief from his symptoms and no longer had yellow, watery loose stool coming from the anus, and did not experience any other adverse reactions. An abdominal radiographic examination revealed no colon dilation or residual feces (Fig. 1c). The patient was discharged to the nursing home and continued to be monitored by a community doctor. Over the next 3 months, the patient did not experience any further incidents of fecal impaction.

## Discussion

FI is most common in the elderly population and is more prevalent with increasing age [11, 12]. The factors contributing to FI in the elderly include cognitive impairment, reduced rectal sensation, poor mobility, chronic constipation, and weakness [13]. FI usually affects the rectum and can lead to serious complications, such as peritonitis, ulcer, intestinal perforation, and bowel obstruction [5, 14].

Abdominal pain, bloating, nausea, and vomiting are common symptoms of FI, but overflow diarrhea is a rare symptom [6, 7]. We encountered a patient who presented with overflow diarrhea as the initial symptom, which may be caused by the blockage of the colon tract by fecal masses. This can result in liquefied fecal masses flowing out of the anus, mimicking diarrhea and leading to misdiagnosis [15]. Despite conservative treatment at the community health service center, the patient’s condition did not improve until he came to our hospital.

According to the primary mechanism of FI, its main treatment options include manual disimpaction, distal or proximal softening procedures, and elution procedures. However, manual disimpaction may cause damage to the anal sphincter and contaminate the environment, while other methods, such as enema, suppositories, and polyethylene glycol solution, can lead to side effects and high risks of falls and cardiovascular accidents in elderly patients [16]. In severe cases, endoscopy and surgery may be necessary, but successful removal of impaction may also be accompanied by serious complications, including bleeding and perforation [8–10]. It is important to carefully consider the best treatment approach on the basis of the individual patient’s needs and condition.

We recently treated a case of fecal impaction with overflow diarrhea using a device called a Disimpactioner. This device has not yet been widely introduced in the field. The Disimpactioner has several advantages. Firstly, it has a smooth and lubricated material that allows it to easily pass through the feces by rotating upwards. The device does not directly contact the intestinal mucosa, which reduces the risk of bleeding and perforation. However, using endoscopic therapy carries the risk of bleeding and perforation for patients with FI [8, 9]. Secondly, the Disimpactioner allows patients to comfortably lie in a lateral position on the bed during treatment, without the need for repeated trips to the bathroom or forceful defecation. This can help prevent falls and cardiovascular accidents in elderly patients. Thomas Sommers *et al*. [3] found that nearly 90% of emergency department visits for fecal impaction required hospital admission, with a minimum stay of 3 days. This device can help patients receive care safely in outpatient and emergency departments, avoiding hospitalization. Additionally, the traditional method of using fingers to remove stool is time consuming and labor intensive. The Disimpactioner’s spiral shape allows for effective force application and is easy to operate, making it suitable for community doctors. Finally, the primary function of the transparent sealing bag is to avert fecal overflow, creating a controlled environment for medical procedures. In the clinical setting, inadvertent fecal spillage poses a common challenge, leading to contamination of patient attire, the surgical bed, and the ambient air in the operating room. The implementation of the Disimpactioner and the transparent sealing bag significantly mitigate the discomfort experienced by patients, fostering an enhanced diagnostic and treatment milieu for healthcare practitioners. This not only renders medical interventions more comfortable, but also underscores a more compassionate approach to patient care.

Despite the practical utility of the Disimpactioner in managing fecal incontinence (FI), it is imperative to acknowledge its inherent limitations. Specifically, it is not suitable for patients with low rectal tumors, inflammatory bowel disease, or mental and behavioral abnormalities. Consequently, a comprehensive evaluation of the patient’s medical history and diagnostic examinations, including radiographic assessments, is essential prior to utilization. In instances in which patients present with conditions, such as malignant hypertension or coronary heart disease, it is advisable to conduct the procedure under electrocardiogram monitoring for added safety.

## Conclusion

FI is a common digestive disease that can have an acute onset. Early identification of FI with atypical symptoms is crucial to provide proper emergency management. In this case, the Disimpactioner was successfully used to relieve the patient’s symptoms. This device is safe, simple, easily employed, and noninvasive, providing a new way of treating FI.

## Data Availability

Not applicable.
